# Fooling with a Panther

**Published:** 1887-05-28

**Authors:** 


### Incidents of Animal Life.

### FOOLING WITH A PANTHER.

This is the title which a New York paper gives to a description of an experience of Silas Smith, an old pioneer, of Wisconsin, when a lad of ten. One day, while he was walking through the woods to the nearest village from his father's clearing, a panther sprang from the limbs of a large beech under which he was passing, and alighted upon the ground beside him. "There was not even a growl," he says, "to warn me of his presence, and he dropped down beside me as softly as a cat and as good-natured as a rabbit. He jumped into the path ahead of me and began to zigzag back and forth across it, purring all the time like a cat, and seeming to be highly pleased when my legs rubbed against him. I was a bit frightened at first, but after a few minutes I made up my mind that it was some animal which would not hurt me, and then we had a romping time. I would clap my hands and shout 'sick 'em!' and the panther would go skurrying here and there like a young pup, sometimes rolling over and over on the leaves, and again making such leaps and bounds as excited my admiration. When we came to a level piece of ground I ran a race with him, and when he got in the path ahead of me I seized the tip of his tail and hung on and laughed until the woods rang. On two or three occasions, as the panther was gambolling about me, he made a clean spring right over my head. It was only when we reached the edge of the village clearing that I suspected the identity of my visitor. The near presence of other human beings seemed to anger him, and he snarled and growled and tore at the earth with his claws. I patted him on the back and smoothed his fur, and he began purring again and was restored to good-nature. He trotted alongside of me about halfway to a barn, about three miles from my father's house, and then slipped away into some fallen timber. I had been quite badly frightened for some minutes, and now seized this chance to make off. My movements were hastened by an ominous growling from the timber, and I walked off at a fast pace, and was within a few steps of the barn when I heard a fierce scream, and turned my head and saw the great cat bounding toward me. The door of the barn was partly open, and without an instant's reflection I sprang inside and closed it. Before I could have counted twenty the panther was at hand. Luckily for me it was a strong door. The beast made terrific efforts to get in, and his sharp claws made the splinters fly as he set them into the wood. When the panther found that he could not get in by the door he sprang upon the roof. I heard him as he alighted on the shooks, and facing about, I saw there was a square hole in the roof, left for a chimney never built. There was nothing to prevent him from leaping down, and this was just what he intended doing. I saw his shadow as he peered over the edge of the opening, and I was all ready as his lithe body alighted on the floor. In two winks I was outside and had the door pulled to, and again he was foiled. He was mad, and no mistake. The eaves of the building were so short that, standing by the door, I could see the ridge-pole. In two or three minutes I heard the clatter of claws as the beast went up the logs, and the moment half his body showed through the opening I changed my position to the inside. He came down the roof, sputtering and growling, and again made a fierce attack on the door. I was too much for him, and he again tried the roof and the chimney-hole. He came down and I went out, and he went up and I came in. He was at the door, snarling in a way to send shivers up and down my back, when I heard the report of a rifle, and directly two men came running up. The shot had been fatal, and the panther was dead. The men were going to the woods to hunt, and, although close at hand, the panther had not noticed them."

*(To be continued.)*

